# Endoscopic-assisted microsurgical resection of giant vestibular schwannoma in semi-sitting position

**DOI:** 10.3171/2021.7.FOCVID2176

**Published:** 2021-10-01

**Authors:** Gang Song, Liyong Sun, Yuhai Bao, Jiantao Liang

**Affiliations:** Department of Neurosurgery and CHINA-International Neuroscience Institute, Xuanwu Hospital, Capital Medical University, Beijing, China

**Keywords:** vestibular schwannoma, semisitting position, facial nerve

## Abstract

The main objectives of microsurgery for vestibular schwannoma are total tumor removal and preservation of facial and cochlear nerve function. For giant tumors, total tumor removal and facial nerve function preservation are challenging. The semisitting position has some advantages. In this video the authors show the removal of a giant vestibular schwannoma with the patient in a semisitting position. They demonstrate the advantages of the semisitting technique, such as the two-handed microsurgical dissection technique and a clear operative field. Finally, a small residual tumor in the internal auditory canal was removed by endoscopy. The patient’s facial function was House-Brackmann grade I at discharge.

The video can be found here: https://stream.cadmore.media/r10.3171/2021.7.FOCVID2176

## Transcript

This video demonstrates endoscopy-assisted microsurgical resection of a giant vestibular schwannoma (VS) in the semisitting position.^1^

### 0:28 Patient Presentation

A 28-year-old female patient presented with a 1-month history of progressive left tinnitus and hearing impairment.

### 0:36 Audiometric Examination

The patient’s facial function was normal before the operation. Audiometric examination revealed hearing loss in the left ear. Magnetic resonance imaging (MRI) showed a homogeneously enhanced tumor in the left cerebellopontine angle, extending into the internal acoustic canal (IAC). The brainstem was compressed by the tumor. The tumor diameter was approximately 4.4 cm, which can be graded as T4b according to the Hannover classification system.^2^

### 1:05 Preoperative CT

Preoperative computed tomography (CT) scan revealed that the IAC was enlarged. No enlarged emissary veins or high jugular bulb was observed in the scan. The x-ray did not reveal cervical instability, indicating a reduced potential risk of neck injury during head positioning. For resection of this type of large tumor, we routinely use the retrosigmoid approach and place the patient in the semisitting position.

### 1:30 Patient Position

The advantages of the semisitting position are as follows: the cerebellum can be relaxed fully after the cerebrospinal fluid (CSF) is released, good brain relaxation can be achieved to optimize the surgical exposure, and a clean and bloodless operative field can be easily obtained in this position. The use of bipolar coagulation for hemostasis can be reduced to avoid damage to nerves.^3–6^ Before surgery, the patient was examined using transesophageal echocardiography (TEE) to rule out patent foramen ovale. Continuous intraoperative monitoring was performed with precordial Doppler echocardiography, TEE, and right atrial catheterization to facilitate early detection and intervention for venous air embolism.

We also used somatosensory evoked potentials (SEPs) and motor evoked potentials (MEPs) of the extremities, facial MEPs, and facial stimulation to monitor neurological function during surgery.

### 2:23 Operative Procedure

We used curved linear incision behind the ear. The margins of the transverse and sigmoid sinuses were exposed. The dura was incised in a C-shaped fashion. After the dura was opened and turned medially, CSF was drained from the cerebellomedullary cistern. Here, we used the facial stimulation polar to stimulate the dorsal side of the tumor to exclude the presence of the facial nerve (FN) on the surface of the tumor. Here, we used a cavitron ultrasonic surgical aspirator (CUSA) for intracapsular debulking of the tumor, creating a working space for the subsequent procedure.

### 3:00 Excision

The dura of the posterior wall of the IAC was removed with arc-shaped excision. The bone was progressively removed with a high-speed diamond drill for exposure of the tumor extending into the IAC. To prevent the nerves from heating, irrigation was necessary to cool the bone during drilling. Here, we also used a stimulator to exclude the presence of FN. In the area of the fundus, we carefully used a right-angle dissector to remove the tumor in a piecemeal fashion. If it is difficult to remove the tumor within the IAC, the posterior wall can be further removed to expose the tumor. The tumor adhering to the FN should be meticulously dissected. Here, we identified the FN by using a stimulator.

### 4:02 Microsurgical Dissection

Microsurgical dissection was carefully performed to dissect the tumor and capsule membrane. We tried to keep the tumor capsule membrane intact for preservation of the FN. Here, we can see that the assistant surgeon continuously irrigates the operative field with saline solution, so that a clean and bloodless operative field can be easily obtained. This helps the primary surgeon avoid using suction to remove the blood and enables dissection of the tumor using two microforceps in both hands. We used the tumor-holding forceps to hold the tumor and another forcep to peel off the capsule membrane from the tumor. During dissection, attention should be paid to the electromyographic response of the FN to prevent injury to the nerve. Constant use of a stimulator to confirm the position of the FN is necessary. The use of CUSA is a quick and easy way to debulk tumors. Here is the root entry zone of the FN on the brainstem. We also identified that the course of the FN was at the anterior-superior side of the tumor. The strongest adherence between the tumor and FN was in the vicinity of the IAC. Therefore, this part of the tumor and capsule must be carefully dissected. Hence, a subcapsular resection of the tumor ensured that the FN was not damaged. In this case, it was difficult to distinguish the FN from the tumor as the FN was dispersed and flattened by the tumor, but the surgical cleavage plane was present. In cases where the FN is membranous and not distinguishable from the tumor and there is no cleavage plane, the surgeon should determine if there is a residual thin layer of tumor on the FN to preserve the FN function. The bleeding site near the FN can be compressed by Gelfoam or Surgicel for hemostasis. Bipolar coagulation should be avoided to prevent any injury to the FN.

### 7:09 Endoscope

Here, we evaluated the IAC using an endoscope and found a small amount of residual tumor within the IAC. We carefully used a right-angle dissector to remove the residual tumor.

### 7:22 Tumor Removal

Finally, the tumor was completely removed, and the FN was intact. The posterior wall of the IAC was sealed using a piece of muscle with fibrin glue.

### 7:36 Postoperative MRI

Postoperative MRI revealed complete tumor resection. The postoperative FN function of the patient was House-Brackmann grade I^7^ at 3 months’ follow-up.

### 7:49 Conclusions

In the semisitting position, a wide and clear operative field can be obtained, and the two-handed microsurgical dissection technique can be employed for VS removal. In addition, the use of bipolar coagulation can be reduced. These advantages are conducive to FN preservation. Endoscopy can help remove residual tumors in the IAC.^8^

## Acknowledgments

This operation follows “Samii’s principle” in vestibular schwannoma surgery. We appreciate the guidance of Prof. Madjid Samii.

## Disclosures

This study was supported by the Beijing Medical Authority’s “Sailing” Plan (XMLX201821).

## Author Contributions

Primary surgeon: Liang. Assistant surgeon: Song. Editing and drafting the video and abstract: all authors. Critically revising the work: all authors. Reviewed submitted version of the work: Liang, Song, Bao. Approved the final version of the work on behalf of all authors: Liang. Supervision: Liang, Bao.
